# Left ventricular dyssynchrony as marker of early dysfunction in premature ventricular contraction-induced cardiomyopathy

**DOI:** 10.3389/fcvm.2022.978341

**Published:** 2022-08-24

**Authors:** Gurukripa N. Kowlgi, Alex Y. Tan, Karoly Kaszala, Michael C. Kontos, Pedro Lozano, Kenneth A. Ellenbogen, Jose F. Huizar

**Affiliations:** ^1^Department of Cardiovascular Medicine, Mayo Clinic, Rochester, MN, United States; ^2^Division of Cardiovascular Diseases, Department of Internal Medicine, Hunter Holmes McGuire Veterans Affairs Medical Center, Richmond, VA, United States; ^3^Division of Cardiovascular Diseases, Department of Internal Medicine, Pauley Heart Center, Virginia Commonwealth University, Richmond, VA, United States; ^4^Department of Cardiovascular Diseases, Medical College of Wisconsin, Milwaukee, WI, United States

**Keywords:** premature ventricular contraction/complex, cardiomyoapthies, dyssynchronopathy, strain imaging, electromechanic

## Abstract

**Background:**

Strain imaging has been suggested as a tool to detect early left ventricular (LV) dysfunction due to frequent premature ventricular contractions (PVCs) in patients with preserved LV ejection fraction (EF). However, the progression of intraventricular dyssynchrony (IVD), radial, and circumferential strain (RS, CS) in PVC-cardiomyopathy (CM) are unknown. The aim of this study was to elucidate the progression patterns of CS, IVD, and electro-mechanical latency (EML) in PVC-CM.

**Methods and results:**

Pacemakers were implanted in 20 canines to reproduce ventricular bigeminy at 200ms (PVCs *n* = 11) for 12 weeks and compared to a sham group (*n* = 9). We obtained echocardiograms at baseline, 4-, 8- and 12-weeks. RS and CS were obtained at the LV mid-cavitary level. IVD was defined as the time between the earliest and latest peak RS. EML was defined as the time between the onset of QRS and the earliest peak RS. LVEF (62 ± 5 to 42 ± 7%, *p* < 0.01), CS (–18 ± 3 to –12 ± 3, *p* < 0.01), and EML (219 ± 37 to 283 ± 46ms, *p* = 0.02) changed significantly in the PVC group. Peak CS (–18 ± 3 to –14 ± 4, *p* = 0.02) and IVD (49 ± 31 to 122 ± 103, *p* = 0.05) had a significant change at 4-weeks despite preserved LVEF (51 ± 5%). IVD normalized while EML increased at weeks 8 and 12.

**Conclusion:**

Our findings consolidate the existing theory that changes in strain precede changes in LVEF in PVC-CM. While IVD becomes abnormal early in the development of PVC-CM, it pseudo-normalizes at advanced stages due to further increases in EML suggestive of cardiac contractility remodeling. These findings are consistent with recent published data where abnormal LV mechanics could be part of a substrate that can predispose to worse outcome in PVC-Cardiomyopathy.

## Introduction

Premature ventricular contractions (PVCs) are the most common arrhythmia affecting the ventricle (1). The burden of PVCs on a 24-h Holter monitor has been shown to be directly proportional to the severity of left ventricular (LV) dysfunction (2–5). Additionally, retrospective studies found a reversal of cardiomyopathy (CM) after PVCs were eliminated (2–10). This led to the description of a reversible disease entity called PVC-CM, which is a diagnosis of exclusion in patients with LV ejection fraction (LVEF) < 50% and frequent PVCs (10). The incidence of systolic dysfunction in patients with frequent PVCs has been estimated at 62.8 per 1000 patient-years over five years (11). Furthermore, PVC-CM, like any CM, leads to heart failure admissions and implantation of defibrillators and resynchronization devices (9, 12).

The mechanisms by which PVCs may induce CM are yet unknown; however, some predominant theories include: (1) abnormal LV mechanics and dyssynchrony (13–16) with subsequent disruption of LV wall motion synergy (4, 17); (2) increase intracellular calcium concentration and myocardial oxygen consumption associated with post-extrasystolic potentiation (18); ^(^3) hemodynamic alterations with remodeling of the autonomic nervous system, and (4) higher mean heart rate (HR) with a similar pathophysiology responsible for tachycardia-CM (19). We have developed a canine model that simulates PVC-CM to allow the mechanistic study of PVC-CM (20). We compared chronic exposure to PVCs with a sham group, to identify crucial differences in patterns of strain measurements. Furthermore, we aim to understand the echocardiographic progression of LV mechanics in PVC-CM, proposing a novel parameter of LV contractility and dysfunction referred to as “electro-mechanical latency” (EML).” EML was defined as the time (ms) between the onset of QRS and the earliest peak radial strain.

## Materials and methods

### Animal model

Twenty mongrel female canines (9–12 months old, weight 22–26 kg) were implanted with a single-chamber pacemaker to reproduce ventricular bigeminy. Through a left thoracotomy under general anesthesia with propofol 1mg/kg (induction) and inhaled isofluorane 2–3%, a bipolar steroid-coated sutureless bipolar Myopore™ (Greatbatch Medical, Alden, NY) epicardial bipolar lead was screwed in the right ventricular (RV) apex for each animal.

After complete surgical recovery, animals were randomly assigned to either persistent 3-month 50% burden of right ventricular (RV) apical PVCs (*n* = 11) at 200ms coupling interval or sham (no ectopy, *n* = 9) using our previously validated premature pacing algorithm (20). The sham group, despite being subjected to a thoracotomy and an epicardial lead implant, was not exposed to any RV pacing (programed ODO). A baseline electrocardiogram without ectopy was performed. Once this initial evaluation was completed, canines started the premature ectopic protocol based on their group assignment, as described above. Echocardiograms without ectopy were repeated at 4- and 8-weeks. The protocol was completed after 12 weeks when final echocardiograms were obtained without ectopy.

This study conformed to the Guide for the Care and Use of Laboratory Animals and was approved by our Institutional Animal Care and Use Committee (IACUC). All animal procedures conform to the guidelines from Directive 2010/63/EU of the European Parliament on the protection of animals used for scientific purposes and the current National Institutes of Health guidelines.

### Echocardiographic evaluation

Echocardiography was obtained with a 5 MHz probe using a commercial system (Vivid-7, Vingmed- General Electric, United States) and at least 80 frames per second using tissue Doppler imaging (TDI) and Speckle-tracking strain analysis software (EchoPAC, GE, United States). Cardiologists with over ten years of clinical experience performed the echocardiograms on canines placed under mild sedation with acepromazine 25–50 mg p.o. 1 h prior to the echocardiogram. The quality of the acquisition process was determined to be adequate.

To assess the chronic effects of frequent PVCs on LV function, we performed echocardiograms at least 15 min after disabling the pacing algorithm. All parameters were obtained following the American Society of Echocardiography guidelines (21). All measurements were performed in at least two different beats and averaged to minimize bias by a cardiologist blinded to randomization group (certified by the National Board of Echocardiography, 5-year echocardiogram experience). LVEF was assessed by biplane Simpson’s formula. LV function was assessed only when the premature pacing algorithm was disabled, and no PVCs or PACs were present.

LV mechanics were evaluated by (1) LV circumferential strain (CS) and radial strain (RS); (2) LV-RV inter-ventricular dyssynchrony; (3) LV intra-ventricular dyssynchrony (IVD) and (4) LV electro-mechanical latency (EML).

Left ventricular (LV), CS, and radial strain (*RS)* values were obtained at the papillary muscle level. Speckle-tracking analysis provided the mean value of peak systolic CS for each LV segment and the peak CS strain for the entire LV (conventionally expressed in negative values) (7, 15). Radial strain and longitudinal strain were not obtained due to the inability to obtain the remaining two different short-axis levels (apical, and mitral valve) and long-axis views (apical 3-chamber) in all animals at different time points.

*Inter*ventricular dyssynchrony was evaluated by the RV and LV electromechanical delay (time from QRS onset to the onset of pulmonary systolic flow and aortic systolic flow, respectively) (22–24).

**FIGURE 1:**
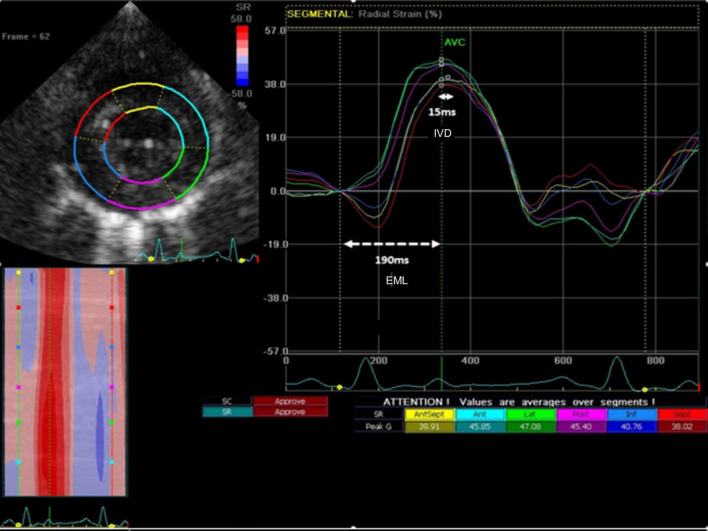
Representative image describing the study measurements with radial strain. Intraventricular dyssynchrony (IVD) was measured as the time from earliest peak segmental radial strain to latest peak segmental strain (solid line – 15 ms). Electromechanical latency (EML) was measured as the time from the onset of QRS to earliest peak segmental radial strain (dashed horizontal white line – 190 ms).

*Intraventricular dyssynchrony (IVD)* and *electromechanical latency (EML)* were evaluated by speckle-tracking strain analysis. IVD was measured as the time between the earliest and latest peak radial strain between the 6 different LV segments (significant IVD defined as time difference ≥130 ms in humans) (7). EML was defined as the time (ms) between the onset of the QRS and the earliest peak radial strain (Figure 1). QRS duration was measured separately at all time-points.

Standard limb ECG leads were obtained peri-operatively at baseline and during final surgery. QRS duration (averaged over three beats) was assessed at baseline and after 12-weeks in both groups.

### Data and statistical analysis

Echocardiographic assessment of LV function in both groups was made without PVCs for accurate calculation of LVEF. The echocardiograms were reviewed by cardiologists blinded to the animal randomization arm.

Continuous variables were expressed as mean ± (SD). Categorical variables were presented as proportions (%) and analyzed using Chi-square (χ^2^) test or Fisher’s exact test when appropriate. Given the small samples sizes, continuous variables were analyzed using Wilcoxon Signed Rank Test. When more than two groups were present, related-samples Friedman’s Two-Way ANOVA or Kruskal-Wallis H tests were used. A *p* ≤ 0.05 was considered as statistically significant. All statistical analysis was performed using SPSS Statistics, Version 27.0 (Armonk, NY: IBM Corp).

## Results

### Echocardiographic data

**FIGURE 2:**
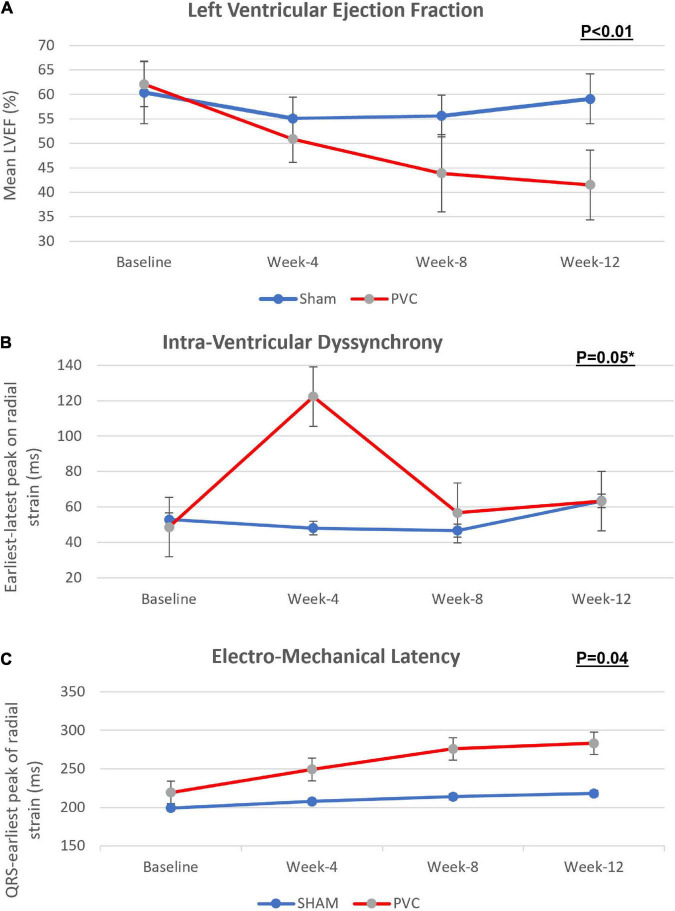
Progression of **(A)** left ventricular ejection fraction, **(B)** intra-ventricular dyssynchrony and **(C)** electro-mechanical latency across bigeminal PVCs and sham groups over 12 weeks. This figure demonstrate that while LVEF decreases gradually over the 12 weeks in the PVC-CM group, an early intraventricular LV dyssynchrony is present that pseudo-normalizes after the remodeling of peak cardiac contractility homogenizes in all segments, only identified by prolongation of EML. PVC group *n* = 11; sham group *n* = 9. Error lines denote standard deviation. (*P* values, Friedman’s two-way ANOVA); * *P* < 0.05 at 4 week.

There was no difference in any parameters at baseline between the PVC and sham groups (Table 1). In contrast to the sham group (59.1 ± 5.1%), LVEF was significantly lower in the PVC group after 12 weeks of bigeminy (41.5 ± 7.1%, *p* < 0.01) (Figure 2 and Table 1).

**TABLE 1:** Progression of echocardiographic parameters during normal sinus rhythm after 12-weeks of persistent ventricular bigeminy.

Group	Sham *N* = 9	PVC *N* = 11	*P*-value (across groups)
**LVEF (%) Mean ± SD**			
Baseline	60.4 ± 6.4	62.1 ± 4.6	0.58
Week-4	55.1 ± 4.4	50.9 ± 4.8	0.002
Week-8	55.6 ± 4.3	43.9 ± 7.9	< 0.001
Week-12	59.1 ± 5.1	41.5 ± 7.1	< 0.001
P-value (within group)	0.1	<0.001	
**Peak global circumferential strain**			
Baseline	–18.8 ± 3.5	–18.8 ± 3.3	0.9
Week-4	–20.1 ± 4.2	–14.1 ± 3.6	0.004
Week-8	–20.9 ± 3.8	–13.2 ± 1.8	<0.001
Week-12	–18.4 ± 3.4	–11.8 ± 3.0	<0.001
P-value (within group)	0.38	<0.001	
**Inter-ventricular dyssynchrony**			
Baseline	30 ± 10.4	27.5 ± 15.8	0.69
Week-12	37.2 ± 15.9	32.3 ± 22	0.37
P-value (within group)	0.28	0.57	
**Intraventricular dyssynchrony**			
Baseline	52.9 ± 31.9	48.7 ± 31	0.94
Week-4	48.1 ± 37.4	122.3 ± 103.4	0.07
Week-8	41.9 ± 37.5	56.7 ± 41.3	0.65
Week-12	63.4 ± 37.5	63.3 ± 34.4	0.95
P-value (within group)	0.59	0.03	
**Electromechanical latency**			
Baseline	199.2 ± 37.8	219.4 ± 36.9	0.29
Week-4	207.7 ± 21.1	249.2 ± 54	0.07
Week-8	214.0 ± 38.3	276.0 ± 45.9	0.009
Week-12	218 ± 36.7	283.2 ± 46.3	0.001
P-value (within group)	0.67	0.01	

Data presented in mean ± standard deviation (P-value, one-way ANOVA within and across groups). PVC, Premature ventricular contraction.

No significant difference in interventricular dyssynchrony (QRS to RVOT and LVOT QRS Vmax duration) was noted over time for each group or across different groups at any time point (Table 1).

**FIGURE 3:**
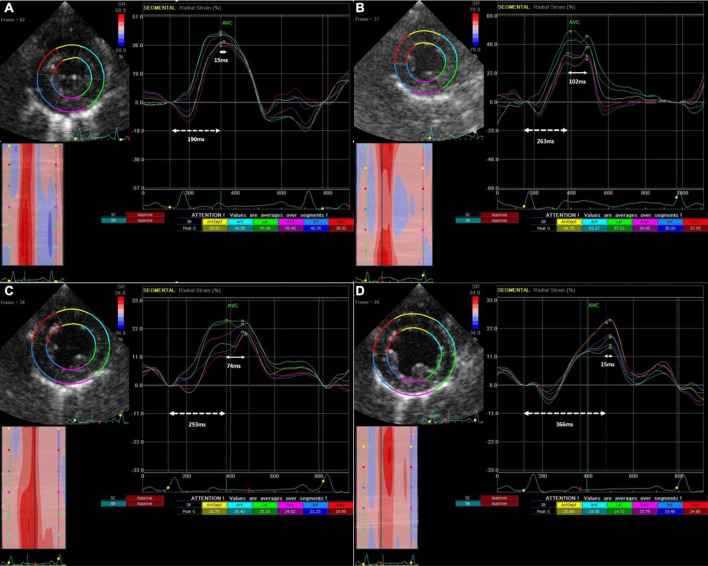
Progression of Intra-ventricular dyssynchrony and Electro-Mechanical Latency from baseline **(A)** to 4 weeks **(B)**, to 8 weeks **(C)** and 12 weeks **(D)** in a single representative animal from the PVC cohort. IVD (solid arrow line) is the time between the earliest and latest peak radial strain amongst different LV segments, while EML (dotted arrow line) is the time between QRS onset and earliest peak radial strain.

*Intraventricular dyssynchrony (IVD) and Electro-mechanical latency (EML)* were found to have an intriguing association. In the sham group, neither IVD nor EML changed over 12 weeks. However, we observed a significant increase in IVD at week-4 in the PVC group (48.7 ± 31 ms to 122.3 ± 103.4 ms, *p* = 0.05) followed by pseudo normalization at weeks 8 (56.7 ± 41.3 ms) and 12 (63.3 ± 34.4 ms). In contrast, EML progressively increased only in the PVC group from 219.4 ± 36.9 ms at baseline to 283.2 ± 46.3 ms at 12 weeks (*p* = 0.02) (Figures 2, 3). Furthermore, EML was significantly higher in the PVC group when compared to sham at week 4 (249.2 ± 54.0), week 8 (276.0 ± 45.9 ms) and 12 (283.2 ± 46.3 ms) (Table 1).

The PVC group had significant worsening in peak CS (–18.8 ± 3.3 at baseline to –11.8 ± 3.0 at week 12, *p* < 0.01) compared to sham (–18.8 ± 3.5 at baseline to –18.4 ± 3.4 at week 12) (Table 1). Interestingly, peak CS (–18.8 ± 3.3 to –14.1 ± 3.6, *p* = 0.02) and IVD (48.7 ± 31 to 122.3 ± 103.4, *p* = 0.05) in PVC group had a significant change at 4-wks despite a relatively preserved mean LVEF (50.9 ± 4.8%).

### QRS duration

Despite the change in EML noted above, the QRS duration of the sinus beat did not have significant change over the 3-month period regardless of the assigned group (QRS duration: PVC 57 ± 6.2 ms to 58.4 ± 8.6 ms, *p* = 0.28 and sham 58.3 ± 6.4 ms to 57.9 ± 6.7 ms, *p* = 0.72).

## Discussion

### Overview and main findings

Premature ventricular contractions (PVCs) are associated with an increased risk of developing CM as supported by an improvement or normalization in systolic function once PVCs are eliminated (3, 4, 11, 19). While PVC burden is clearly an important parameter that determines PVC-CM, why some patients do not develop PVC-CM despite a high burden is unclear. Researchers have identified some factors that may help in risk-stratifying patients including QRS duration of PVC, right ventricular PVCs, Del Carpio Munoz et al. (25) coupling interval, Sun et al. (26) dyssynchrony as measured by strain, Yao et al. (15) or location of PVCs and dyssynchrony measured through hemodynamics (16). While some of these factors, such as QRS duration, are surrogates of dyssynchrony and may have other causal factors, others such as hemodynamic measurements are invasive and may not be practical to measure in all patients presenting with frequent PVCs. Our study describes the natural progression of PVC-CM with easy to measure echocardiographic parameters that if reproduced in human studies, may be able to identify patients at higher risk of PVC-CM.

Our main findings include: (1) Myocardial strain can identify early stages of PVC-CM particularly when LVEF is still relatively preserved; (2) Dyssynchrony may be an indicator of early-stage PVC-CM with frequent ventricular ectopy; (3) PVC-CM is characterized by an early intraventricular dyssynchrony that pseudo-normalizes after remodeling of cardiac contractility in all segments, only identified by prolongation in EML.

### Strain and premature ventricular contraction-cardiomyopathy

Our study confirms the development of CM over time with frequent ventricular ectopy. However, reduction in LVEF occurs only after a sizeable portion of the myocardium has been affected. Strain imaging hence has emerged as a critical adjunct in the assessment of systolic function, especially in situations where the LV function is expected to progressively deteriorate (27). The use of strain imaging, especially global longitudinal strain, has been adopted in predicting the development of systolic dysfunction in chemotherapeutic cardiotoxicity (28), valvular heart disease (29, 30), and hypertrophic CM (31). The application on strain imaging in PVC-CM has not been studied in detail. Few clinical studies have described a worsening radial and circumferential strain in patients with frequent PVCs despite preserved LV function (LVEF > 50%) (15). Furthermore, Wijnmaaleen et al. demonstrated that the strain pattern normalizes after PVCs ablation, supporting a mild form of PVC-CM that can only be identified by speckle tracking (32). This current PVC-CM translational study adds to that existing knowledge by temporally comparing CS to LVEF and noted that changes in circumferential strain were observed prior to the decline in LVEF.

### Intra-ventricular dyssynchrony and electromechanical latency

Left ventricular (LV) dyssynchrony has garnered attention as a potential cause of PVC-CM (13–15). Few pre-clinical studies have shown that chronic states of bigeminal PACs do not produce CM when compared to PVCs in swine and canine models (16, 33). Furthermore, it appears that bigeminal PVCs led to a significantly lower LVEF when compared to chronic RV apical pacing at 140 bpm. Thus, it is proposed significant differences in QRS duration and LV dyssynchrony during PVC and confirmed molecular changes of calcium handling proteins that accompany the clinical changes (16, 34, 35). While it can be difficult to justify invasive hemodynamic measurements, particularly in asymptomatic patients, serial echocardiograms to assess strain may be a reasonable tool. To our knowledge, this is the first study describing the echocardiographic progression of LV mechanics during the development of PVC-CM by comparing chronic states of PVC and sham groups. It is important to understand that while IVD assesses the differences in time to peak contraction between different segments of the LV, EML assesses the earliest time from QRS to peak contraction regardless of the LV segment. This important concept is shown in this manuscript, where there could be abnormal contractility reflected only by increased EML despite the lack of or minimal IVD.

The intra-ventricular dyssynchrony findings give rise to an intriguing observation. In the PVC group, IVD becomes abnormal early in the pathogenesis of PVC-CM along with EML. EML progressively increases over the 12 weeks while IVD pseudonormalizes at weeks 8 and 12 suggesting remodeling of cardiac contractility in all segments. In the early weeks of PVC-CM, EML in some myocardial segments are affected to a greater extent than the others, leading to abnormal IVD. However, over time, the EML increases homogeneously between the myocardial segments, leading to pseudonormalization of IVD. This suggests that CM is progressing as the EML continues to lengthen due to myocardial remodeling. Moreover, the lack of changes in QRS duration overtime suggests that alterations in the excitation-contraction coupling, rather than electrical or contractility remodeling alone is likely responsible for EML. Thus, EML rather than IVD may be a novel parameter to assess LV contractile remodeling. PVC-CM appears to have a distinct spectrum of echocardiographic changes, and the combination of EML and IVD may prove useful for identifying where a patient lies in the spectrum to aid in their management. If these findings are corroborated in humans, it would support early intervention for PVCs suppression (either antiarrhythmics or catheter ablation) or at least a close follow up despite a normal LVEF due to a subclinical or early stage of PVC-CM. Our present study required the absence of PVCs to assess the abnormal LV mechanics in PVC-CM. which may not be feasible clinically in patients with frequent PVCs. In such scenarios, assessment of LVEF even immediately after PVCs (post-extrasystolic potentiation) described by our group may also be a useful approach to assess the presence of cardiomyopathy in these patients (36).

A recent study reported abnormal LV mechanics in a PVC-cardiomyopathy swine model (37). Nevertheless, this study did not assess the progression of LV mechanics over time since dyssynchrony was obtained only at baseline and during final surgery (week 12) with pressure-volume loop using a pressure sensing conductance catheter. Moreover, the QRS to earliest peak contraction (EML) was not reported. In contrast to this study, our findings show the presence of LV dyssynchrony at 4 weeks that pseudonormalizes at week 12 due to a homogenization of the EML across LV segments. The differences between studies could represent species differences, however, most likely represents differences in techniques (RS and CS assessed synchrony between opposing walls, while pressure conductance catheters assess dyssynchrony in the long axis of the LV) and sedation, since echocardiograms to assess LV mechanics were performed while awake with minimal sedation (see methodology). Regardless, it is clear that PVC-cardiomyopathy has abnormal LV mechanics that could potentially be part of a substrate that can predispose to worse outcomes (38, 39).

We found interventricular dyssynchrony assessed by RV and LV electromechanical delay was no different between groups over time (22–24). This parameter represented the time taken by each ventricle to result in peak flow in the respective outflow tracts. One potential explanation for this finding could be that in sinus beats, PVC-CM affects the contractile function of RV as much as the LV; hence, no apparent differences in inter-ventricular dyssynchrony were found.

### Limitations

We acknowledge that while an animal model allows the control of several variables, this has inherent limitations, and the study findings may be different in humans. In addition, the number of canines was relatively small; however, the study clear scientific rigor since each animal serve as its own control. Thus, further clinical studies should be performed to confirm and further understand the implications of EML in the early stages of cardiomyopathy. Our pacing methodology simulates PVCs arising from the RV apex, while in clinical practice, PVCs can arise from a multitude of locations that were not directly studied in our model. In addition, we cannot evaluate changes in IVD from apical to basal segments due to the lack of longitudinal strain assessment. As noted on methodology, global strain values were not obtained due to the inability to obtain all required views in all animals at different time points. Finally, right ventricular function in canines cannot reliably be evaluated using echocardiography. Thus, we only evaluated LV function and mechanics, as demonstrated in the clinical setting and other animal models.

## Conclusion

Myocardial strain assessment is an important tool that can identify the early stages of PVC-CM when LVEF is still preserved. Furthermore, PVC-CM may have an early intraventricular LV dyssynchrony that may pseudo-normalize after the remodeling of cardiac contractility homogenizes in all segments, only identified by prolongation of EML. EML is a new term and measurement that could identify a subclinical cardiomypathic state despite a pseudonormal IVD. EML should be evaluated as a part of the assessment of LV mechanics as this may identify contractile remodeling overlooked by IVD in PVC-CM and possibly in other cardiomyopathies. This is supportive of recent data where abnormal LV mechanics could potentially be part of a substrate that can predispose to worse outcome in PVC-Cardiomyopathy. Further studies will be important to see if these findings can be validated in other animal cardiomyopathic models as well as in humans.

## Data availability statement

The original contributions presented in the study are included in the article/supplementary material, further inquiries can be directed to JH, jose.huizar2@va.gov.

## Ethics statement

The animal study was reviewed and approved by McGuire VA Medical Center IACUC.

## Author contributions

JH provided the idea and technical guidance, data analysis, and revised the manuscript. GK provided the data collection and analysis and wrote the manuscript including generation of tables and figures. KK, AT, KE, MK, and PL provided guidance in data analysis and revised the manuscript. All authors reviewed and approved the manuscript.

## Abbreviations

CM: Cardiomyopathy
CS: Circumferential strain
EF: Ejection Fraction
EML: Electromechanical latency
HR: Heart rate
IVD: Interventricular dyssynchrony
PVC: Premature Ventricular contraction
PVC-CM: PVC-Cardiomyopathy
RS: Radial strain
TDI: Tissue Doppler imaging.
